# Assessing housing exposures and interventions that impact healthy cities: a systematic overview of reviews

**DOI:** 10.1177/17579139231180756

**Published:** 2023-08-05

**Authors:** GC Richards, J Carpenter, E Okpalugo, DJ Howard, C Heneghan

**Affiliations:** Centre for Evidence-Based Medicine, Nuffield Department of Primary Care Health Sciences, University of Oxford, Oxford, UK; Global Centre on Healthcare and Urbanisation, Kellogg College, University of Oxford, Oxford OX2 6PN, UK; Centre for Evidence-Based Medicine, Nuffield Department of Primary Care Health Sciences, University of Oxford, Oxford, UK; Global Centre on Healthcare and Urbanisation, Kellogg College, University of Oxford, Oxford, UK; Oxford University Department for Continuing Education, University of Oxford, Oxford, UK; Centre for Evidence-Based Medicine, Nuffield Department of Primary Care Health Sciences, University of Oxford, Oxford, UK; Global Centre on Healthcare and Urbanisation, Kellogg College, University of Oxford, Oxford, UK

**Keywords:** housing exposure, housing intervention, systematic scoping review, healthy cities

## Abstract

**Aim:**

There are direct links between housing and health. However, there is a lack of systematic reviews that bring together the evidence to outline the health impacts of exposures in housing and housing interventions. This article aims to address this gap by synthesising systematic reviews on the themes of housing exposures and interventions.

**Methods:**

We searched four databases: Scopus (Elsevier), PsycINFO (OvidSP), Science Citation Index and Social Science Citation Index (Web of Science Core Collection), and the Sociology Collection (Proquest). We used keywords related to ‘health’ and ‘city*’ and included all types of reviews. We extracted data into a predesigned extraction form and synthesised information narratively.

**Results:**

745 articles were identified and screened, of which 256 reviews were included and 16 (6%) related to housing. All reviews related to housing exposures found that poor housing, including crowding, coldness, dampness, mould, and indoor air pollution had a negative impact on health. Most reviews found that housing interventions such as housing refurbishment, heating, and energy efficiency interventions positively impacted health outcomes. An online toolkit was developed to disseminate and communicate this research: https://www.healthycitiescommission.org/toolkit/.

**Conclusion:**

Governments have a pivotal role in addressing health issues related to housing interventions and exposures in housing. This includes interventions through building regulations following international guidance and financial assistance to encourage housing modifications that will improve health.

## Introduction

Cities are currently facing increasing health inequalities. For example, in the UK, healthy life expectancy (HLE) at birth between the most-deprived and least-deprived areas differs significantly. Between 2018 and 2020, the difference was 18.2 years for men and 18.8 years for women.^1^ These widening health disparities are due to various factors, including the wider determinants of health.^2,3^

There is growing recognition that within these broader determinants, spatial planning plays a critical role in addressing health inequalities.^4^ The configuration of the built environment impacts the key drivers of health, including housing quality and the natural environment, such as green and blue space, and influencing lifestyle factors such as active travel and healthy food choices.^5^

One area of critical interest in this field is housing, as highlighted by the report of the Commission on Creating Healthy Cities.^6^ As the report quotes, ‘*Health is made at home*’,^7^ and the UK Government’s levelling up white paper also reiterates: ‘*Having a decent home is fundamental to our wellbeing*’ (p. 221).^8^

While considerable evidence supports the links between housing and health, the evidence is disparate, and an overview of reviews has not systematically addressed this gap. Therefore, a scoping review of the evidence is required to provide a holistic view of housing issues that affect healthy cities in terms of exposure, interventions and considering broader issues such as the climate emergency and the structural issues of poverty and inequality.

In this review, the definition of ‘healthy cities’ was obtained from the World Health Organization (WHO):

*A healthy city is one that continually creates and improves its physical and social environments and expands the community resources that enable people to mutually support each other in performing all the functions of life and developing to their maximum potential.* ^9^

The aims of this review were to fill the evidence gaps by drawing together the most up-to-date reviews on housing and healthy cities. This article reports a sub-section of a wider review conducted in 2021 by the listed authors for the Commission on Creating Healthy Cities.^6^ The wider review aimed to systematically scope the academic literature for exposures and interventions that affect the creation of healthy cities more broadly.^6^ Housing was one of 12 themes identified in the wider review, with findings from the other themes to be subsequently published. The final aim of the scoping review was to develop an evidence bank (‘toolkit’) that brought together evidence to support practitioners, policymakers, and the public in improving the health of the population in their cities, reporting on the implications for policy and highlighting the gaps where research is urgently needed (https://www.healthycitiescommission.org/toolkit/).

## Methods

We designed a systematic scoping review and preregistered our study protocol.^10^ The Preferred Reporting Items for Systematic reviews and Meta-Analyses extension for Scoping Reviews (PRISMA-ScR) reporting guideline was used to write our article.^11^

### Search strategy

We searched four databases: Scopus (Elsevier), PsycINFO (OvidSP), Science Citation Index and Social Science Citation Index (Web of Science Core Collection), and the Sociology Collection (Proquest) on 16 April 2021. We used keywords related to ‘health’ and ‘city*’ as outlined in Supplement 1. We restricted the search to reviews (e.g. systematic reviews, literature reviews, and all forms of evidence synthesis) and included studies published between January 2000 and April 2021.

### Study screening and eligibility

We included reviews exploring any factor(s) or intervention influencing urban health. For example, a review may have examined the health outcomes after exposure to air pollution or the effectiveness of nutritional or exercise programmes in the workplace. One study author (G.C.R.) screened titles, abstracts, and full texts, resolving uncertainty for inclusion with a second author (C.H.).

### Data extraction and analysis

One study author (G.C.R.) extracted data from included reviews into a predesigned extraction form. Findings were descriptively and narratively summarised based on their impact, resource implications, and the quality of the evidence, and quantitative estimates were reported where feasible and relevant. The criteria used to grade impact, resource implications, and the quality of the evidence are summarised in Supplement 2.

All study materials are available on the Open Science Framework.^12^

## Results

**Figure 1:**
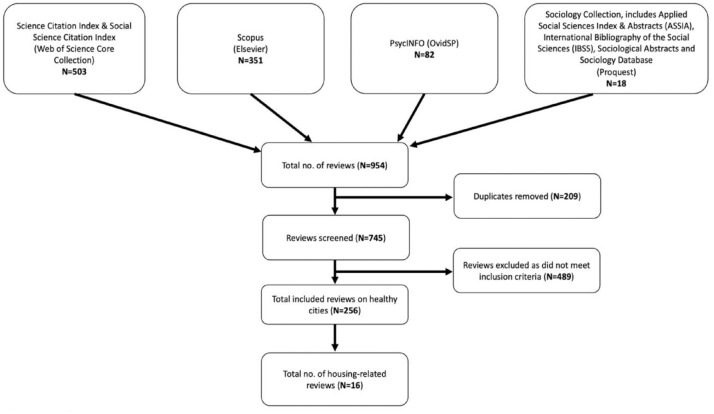
PRISMA flow diagram of the searches conducted on 16 April 2021 and the screening of eligible reviews

The systematic search identified 954 articles, and after the screening, 256 reviews were included (Figure 1). Across the 256 reviews, evidence was grouped into 12 categories, as follows:

- Demographics
- Economics and finance
- Food and nutrition
- Governance and policymaking
- Housing
- Mobility and transport
- Nature
- Security
- Technology
- Urban development
- Urban environment
- Utilities and infrastructure

There were 16 reviews that mentioned housing interventions and exposures related to health and wellbeing, which are discussed and summarised in this article.

### Exposures in housing

Ten reviews examined housing environments’ health impacts,^13–22^ including more than 500 primary studies. Most reviews considered all age groups or did not specify age, with one review focusing on adolescents.^13^ Half restricted their geographical scope, with two focusing on the USA,^14,15^ Brazil,^16^ Europe,^13^ and Sub-Saharan Africa.^17^ The rest did not restrict the evidence geographically, with the highest proportion of studies being from North America, Europe, and Oceania.

Overall, exposure to poor housing was negatively associated with communicable and non-communicable diseases, poor physical and mental health, and mortality. The most frequently reported impacts on health in housing were coldness, dampness, mould, and poor indoor air quality due to a lack of adequate ventilation.

There were associations between housing characteristics and particular diseases. Crowding was associated with a greater risk of infectious and respiratory disease and poor mental health.^17–20^ Damp or mouldy housing was associated with respiratory disease, meningococcal infection, eczema, asthma, and rhinitis.^17–19^ In children, the odds of asthma and asthmatic symptoms, such as wheezing and cough, are two or more times greater in damp houses than in non-damp ones.^17^ Toxic materials used in housing construction or cleaning, such as lead, asbestos, indoor allergens, ozone, and radon, were injurious to health, including cognitive disabilities, neurodevelopmental defects, asthma, cancer, asbestosis, and death.^15,17–19^ Daily cleaning activities were associated with the prevalence of asthma or asthmatic symptoms, with evidence of the chemicals in cleaning agents reducing lung function in women who regularly used cleaning products in the home and children exposed to household air cleaners that were ozone emitting.^19^ Cold or low temperatures were associated with respiratory infections, hypothermia, bronchospasm, and heart disease.^18^ Homelessness was associated with a range of physical ailments, causing ill health, and aggravating poor health.^18^

Poor housing design predisposed residents to accidents and injuries, which increased in children and the elderly.^18^ Features such as a lack of shared recreational space, private gardens, or housing with deck access were found to have a negative impact on mental health.^21^ Defective walls were conducive to the survival of disease-hosting rodents, such as rats and mice, which increased the prevalence of pest-borne diseases, particularly Lassa fever.^17^

Indoor household air pollution from multiple sources (e.g. biomass and solid fuels used for cooking and heating) negatively impacted health, including eye infections, respiratory-related diseases and deaths, cancers, and hypertension.^17,22^ Thus, kitchens using liquefied petroleum gas (LPG) or electric stovetops are better for health,^22^ although indoor nitrogen dioxide concentrations should be monitored.^19^ For children and adolescents, tobacco smoking in the home increased the risk of respiratory disease later in life.^19^ Allergens from pets lead to exacerbation of asthma and wheezing, with evidence of long-term negative impacts of early life exposure on the respiratory system.^19^

In low- and middle-income countries, poor quality housing was associated with disease incidence and vector abundance, including malaria, leishmaniasis, Chagas disease, and schistosomiasis.^16,20^ Living conditions, such as small living spaces and lack of air conditioning, increased dengue transmission in the US–Mexican border area.^20^

Housing insecurity and lack of housing affordability was a psychosocial stressor that affected both physical and mental health. High utility bills required lower-income families to choose between housing, heating, food, medical care, and other basic needs, which negatively affected the growth and development of children.^15^ Residential relocation because of the lack of affordable housing caused a disruption in healthcare.^15^ Housing status was a predictor of pain for people with chronic pain, due to the stress burden of homelessness or living in a low-income neighbourhood.^14^ Housing circumstances (e.g. housing tenure and single-parent households) were determinants of adolescent health and wellbeing.^13^

This evidence was graded for its impact on health, resource implications, and quality of evidence. Overall, exposures in housing had a mild negative impact based on uncertain evidence, with uncertain resource implications. Such grades are described and disseminated in the Healthy Cities Toolkit: https://www.healthycitiescommission.org/toolkit/exposures-in-housing/.

### Housing interventions

Six reviews examined the health impact of housing interventions,^23–28^ involving more than 200 primary studies. Interventions involved changing area characteristics,^23^ structural refurbishments and modifications,^24,25^ provision of adequate heating, improvements to ventilation and water supply, initiatives for prioritising housing for vulnerable groups,^25^ and in low- and middle-income countries, the upgrading of slums^26,27^ and informal settlements.^28^ Overall, housing interventions decreased communicable and vector-borne diseases and improved general health, mental health, and wellbeing.

Most reviews considered all age groups or did not specify age. One review restricted its geographical scope to South Africa^28^ and another to low- and-middle-income countries.^26^

In one review, housing refurbishment and modifications, provision of adequate heating, and improvements to ventilation and water supply were associated with improved respiratory outcomes, quality of life, and mental health.^25^

Interventions to improve deprived areas via employment, training, housing, crime reduction, environment, and quality of life positively improved standardised mortality rates.^23^ Prioritising housing for vulnerable groups also led to improved wellbeing.^25^ Heating and energy efficiency interventions positively impacted general health, respiratory health, and mental health outcomes.^23^

Modifying ceilings to close eaves, installing mosquito-trapping systems, screening windows and doors, and netting covering gable ends reduced indoor vector densities of *Aedes* and *Anopheles* mosquitoes and had a significant effect on the incidence of clinical malaria in southeast Asian homes.^24^

In informal housing in low- and middle-income countries, providing homes with cement flooring was associated with improved maternal mental health and satisfaction with the quality of life.^27^ Slum upgrading had a positive impact on rates of communicable disease with a lower diarrhoeal incidence in intervention groups, which was associated with a lower incidence of nutritional deficiencies and a positive impact on general health and wellness.^27,28^ In informal settlements, upgrading housing types from ‘shacks’ to ‘subsidised housing’ was associated with improvements in noise, violent crime, safety, and reductions in alcohol and substance use.^28^

This evidence was graded for its impact on health, resource implications, and quality of evidence. Overall, housing interventions had a moderate positive impact based on low quality evidence, with moderate resource implications. Such grades are described and disseminated on the Healthy Cities Toolkit: https://www.healthycitiescommission.org/toolkit/housing-interventions/.

## Discussion

Across all included reviews investigating healthy cities, 6% evaluated housing exposures and interventions. All reviews found that poor housing, including crowding, poor structures and design, coldness, dampness, mould, toxicants, and indoor air pollution had a negative impact on health. Several reviews found that housing interventions positively impacted health outcomes.

These findings mirror other research looking at healthy housing, in particular recent work on healthy urbanism that has been published after the completion of the systematic search for this review.^29^

A limitation of the study was that the quality of the evidence, particularly for exposures, was uncertain, as the majority (80%) of reviews did not use tools to assess the risk of bias or quality. There was also considerable study design heterogeneity, which limits comparability. Furthermore, most included primary studies of reviews were cross-sectional, meaning that the direction of causation and effect over time could not be identified. However, the work presents a thorough systematic overview of reviews of one of the most important determinants of health, and therefore represents an important contribution to understanding housing exposures and interventions that impact on healthy cities.

This review points to a number of recommendations that are critical for governments and other stakeholders to implement, to address health through housing. Our findings suggest that governments should ensure adequate ventilation and strong thermal performance standards in housing, including heating, cooling, and insulation, as well as ensuring that these interventions are energy efficient. Also key for healthy housing are smoke-free homes and clean fuels such as LPG or electricity. Government subsidies for clean fuels in homes would promote more significant usage with positive implications for residents’ health.

The evidence suggests a strong rationale for developing and implementing building regulations incorporating international guidance that supports health and wellbeing. In addition, governments should provide financial incentives and assistance to homeowners and landlords to modify housing to improve health. There are also benefits from collaboration in this area, between business, academia, and the public sector, to support research in the field and encourage implementation.

The synthesised evidence also points to the importance of health promotion agendas that focus on infrastructural improvements, access to education, healthcare, and access to essential water and sanitation in low- and middle-income countries. Addressing a person’s housing status and poverty is essential to preventing and addressing chronic diseases.

A key implication of the findings of the housing exposure evidence was the need for collaborations between housing and health agencies to join up agendas and holistically address healthy housing. This could be implemented effectively through partnerships between the public and private sectors.

Future research could focus on gathering more robust evidence that uses larger sample sizes and a longitudinal design to understand the longer-term health impacts of housing exposures and interventions. Moreover, standardised definitions of housing exposures, health outcomes, and housing quality measures are required to improve the ability to synthesise such evidence.

## Supplemental Material

sj-docx-1-rsh-10.1177_17579139231180756 – Supplemental material for Assessing housing exposures and interventions that impact healthy cities: a systematic overview of reviews Supplemental material, sj-docx-1-rsh-10.1177_17579139231180756 for Assessing housing exposures and interventions that impact healthy cities: a systematic overview of reviews by GC Richards, J Carpenter, E Okpalugo, DJ Howard and C Heneghan in Perspectives in Public Health

sj-xlsx-2-rsh-10.1177_17579139231180756 – Supplemental material for Assessing housing exposures and interventions that impact healthy cities: a systematic overview of reviews Supplemental material, sj-xlsx-2-rsh-10.1177_17579139231180756 for Assessing housing exposures and interventions that impact healthy cities: a systematic overview of reviews by GC Richards, J Carpenter, E Okpalugo, DJ Howard and C Heneghan in Perspectives in Public Health
