# Supplementary material for: Assessing housing exposures and interventions that impact healthy cities: a systematic overview of reviews
Source: Perspect Public Health. 2023 Aug 5;145(5):272–6. doi: 10.1177/17579139231180756 (PMC12457718; doi:10.1177/17579139231180756)
Supplement: sj-docx-1-rsh-10.1177_17579139231180756 – Supplemental material for Assessing housing exposures and interventions that impact healthy cities: a systematic overview of reviews [file sj-docx-1-rsh-10.1177_17579139231180756.docx]

**Supplement 2:** Criteria for impact, resources, and quality of evidence

## **What do we mean by ‘Impact’?**

The impact is the effect that an urban intervention has on individual health or the broader wellbeing of society. The impact was assessed using the reported outcomes in the review.

The impact is rated as positive or negative. Uncertain impact indicates that the evidence suggests that the intervention or exposure makes no difference, or there is still considerable uncertainty about the overall effect.

| **Score** | **Parameters*** |
| --- | --- |
| 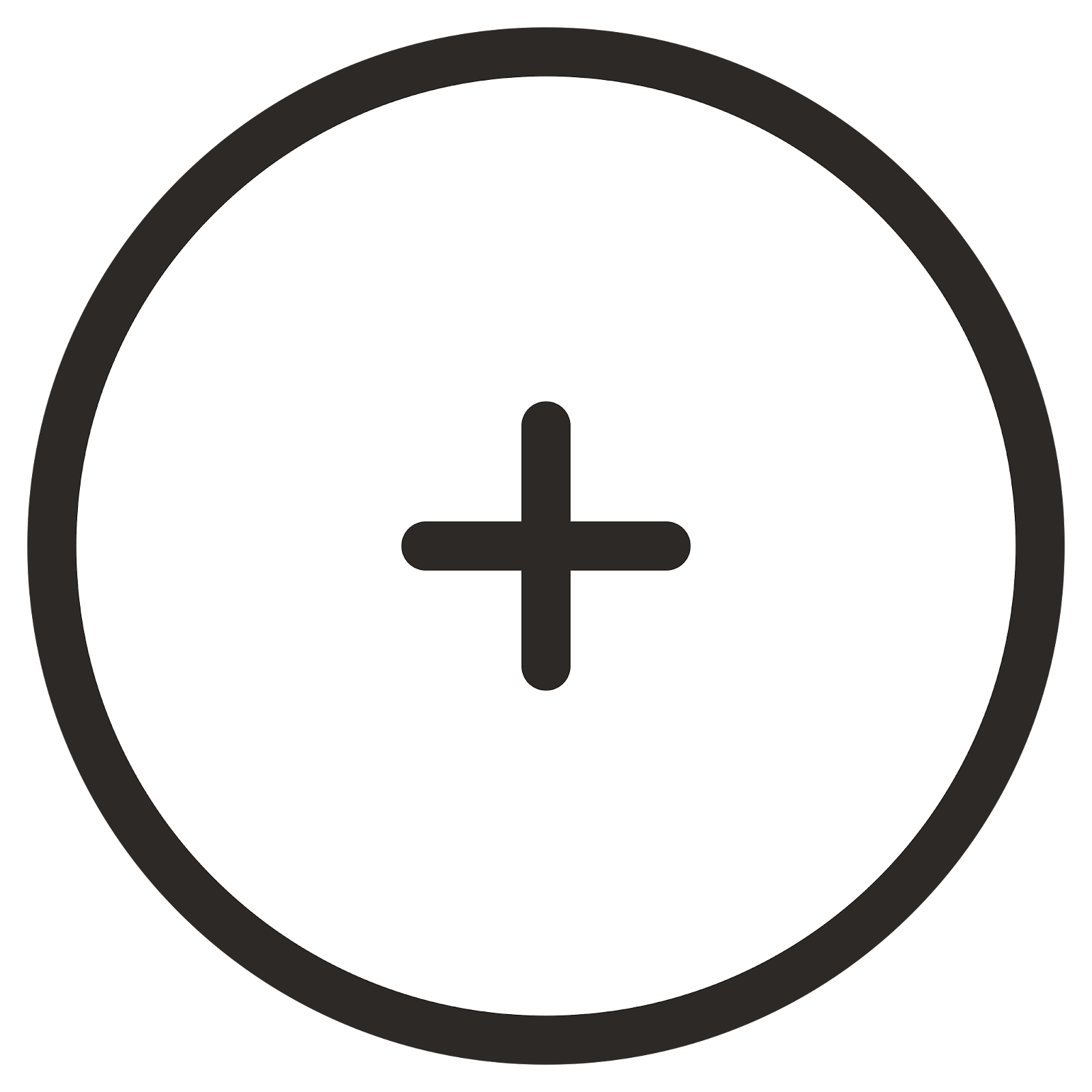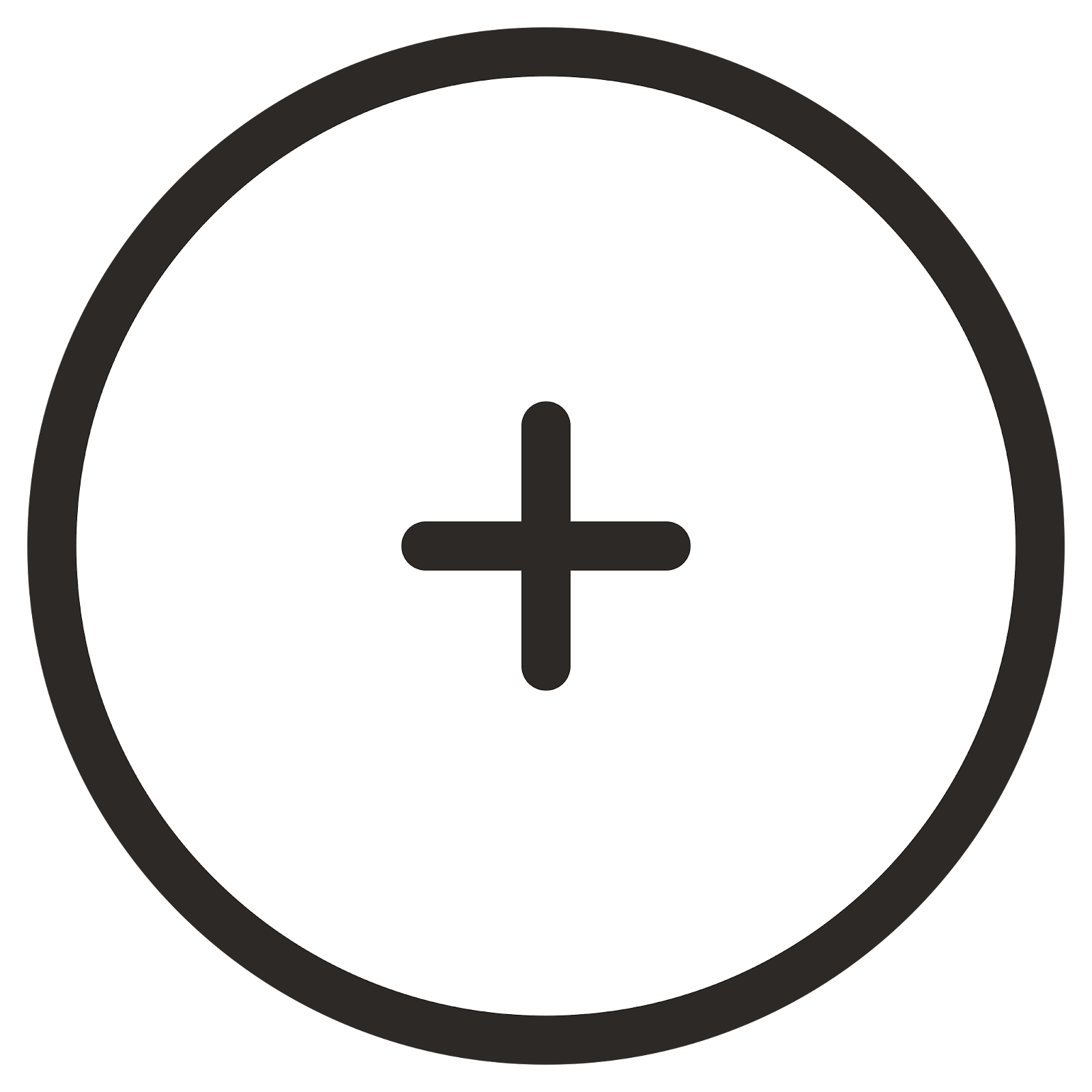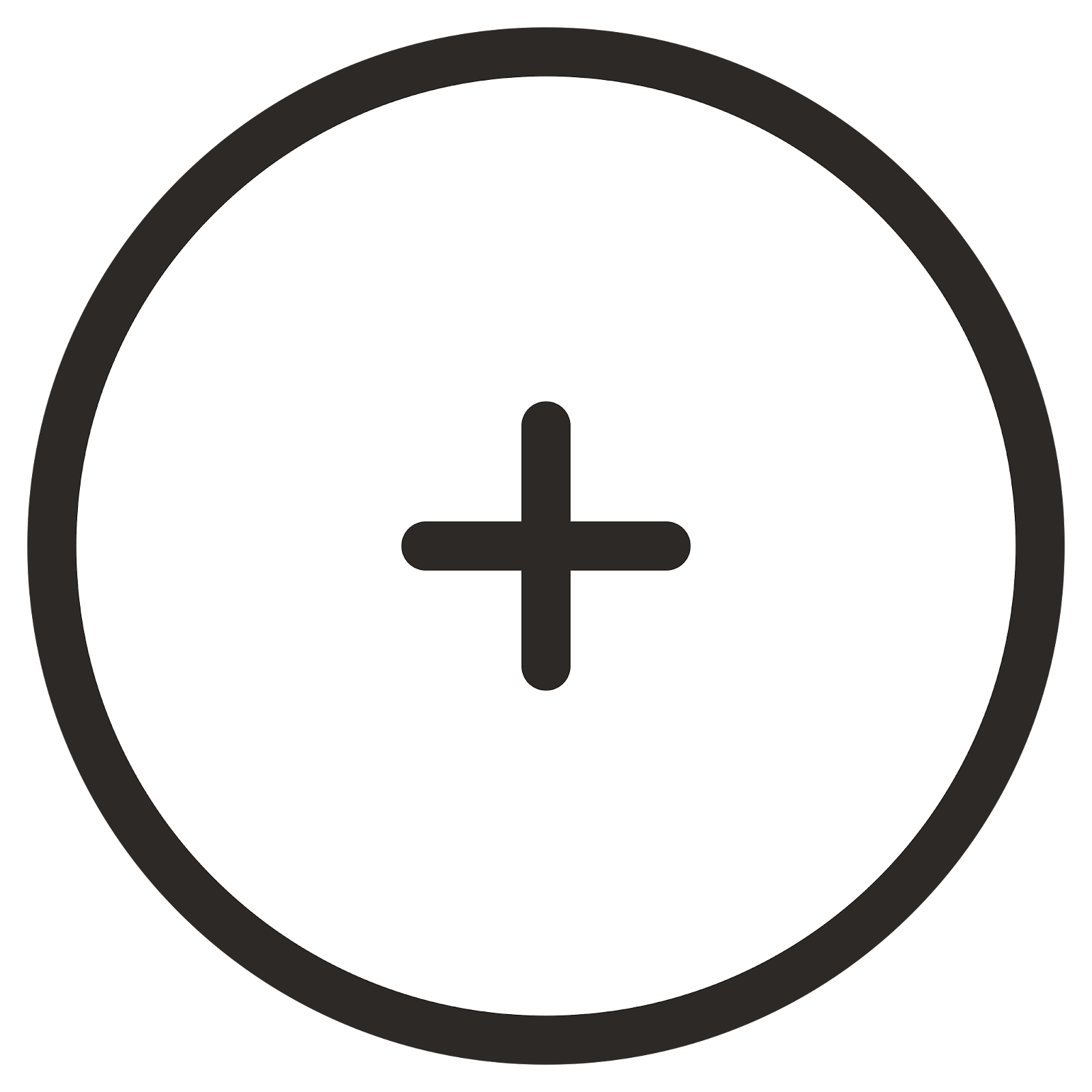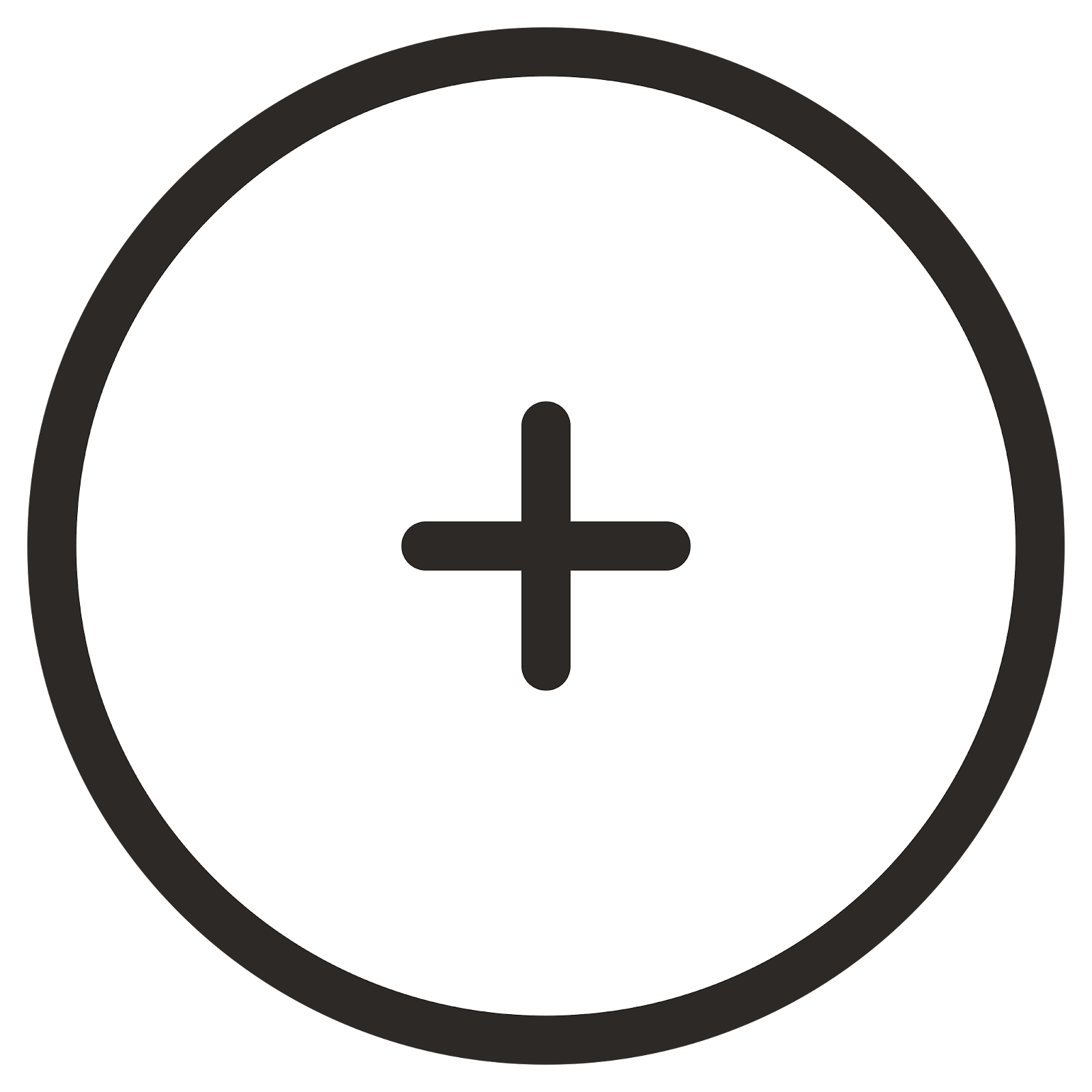 | **Strong positive impact:** Statistically significant effect in >75% of studies or pooled meta-analysis  AND  Clinically significant effect on health in >75% of studies or pooled meta-analysis |
| 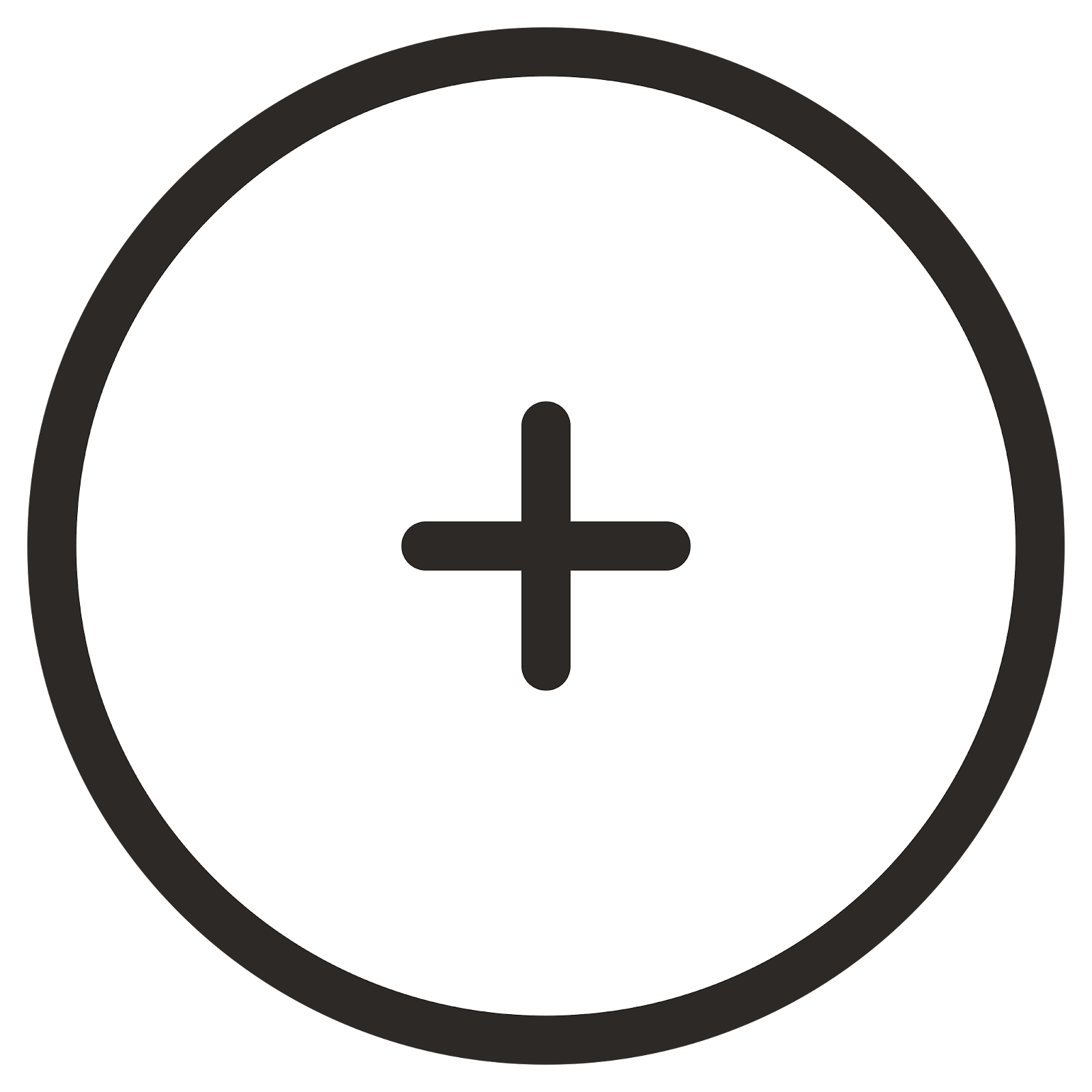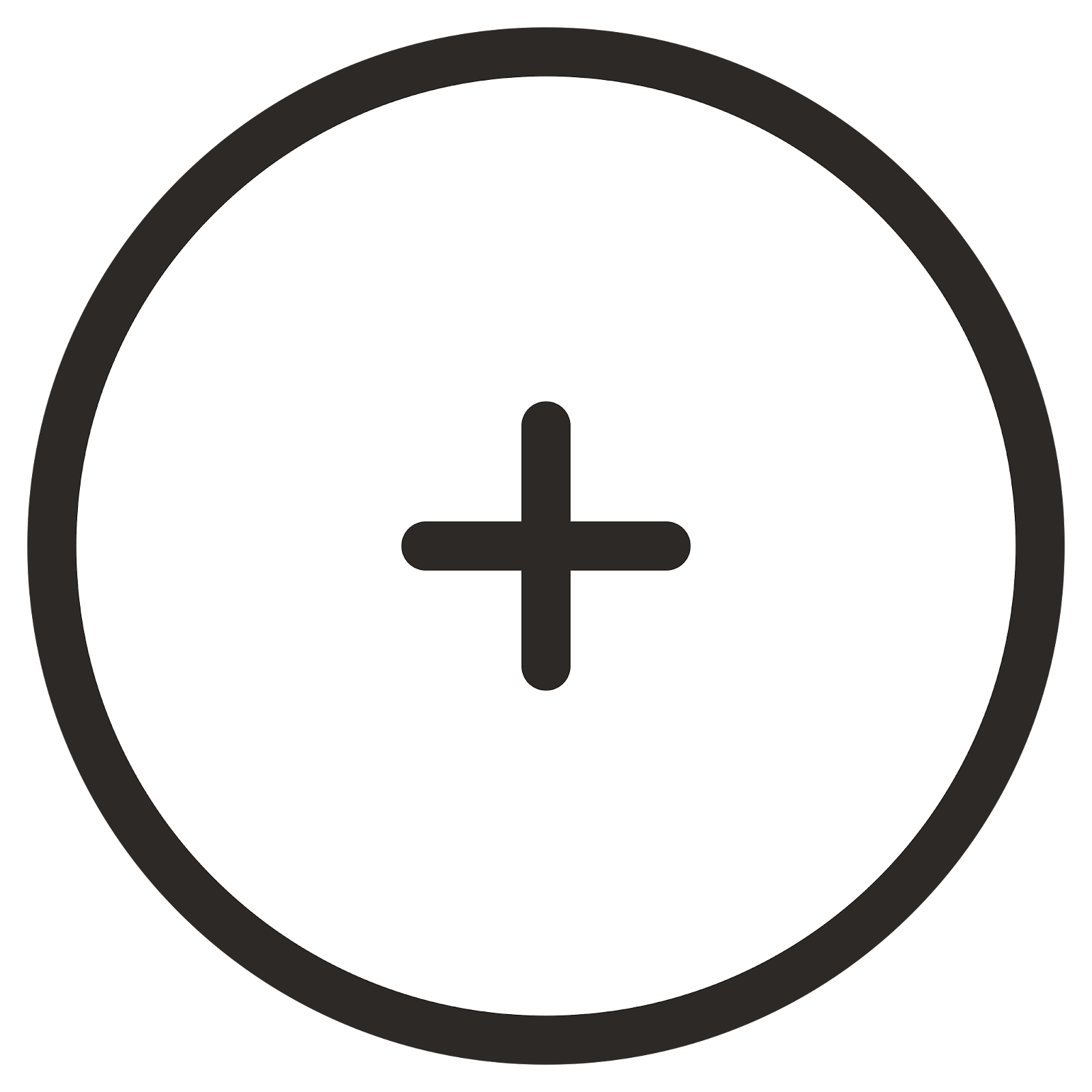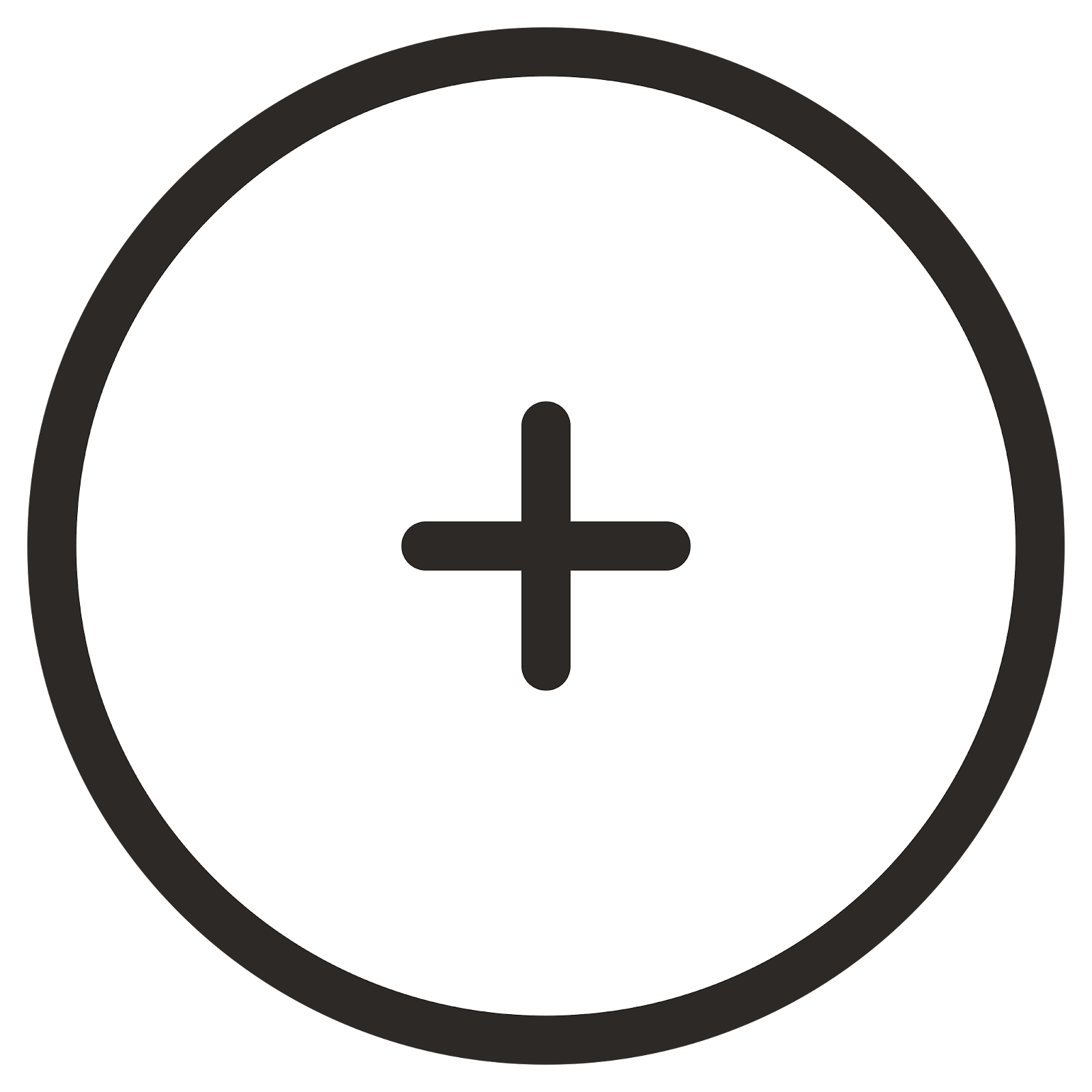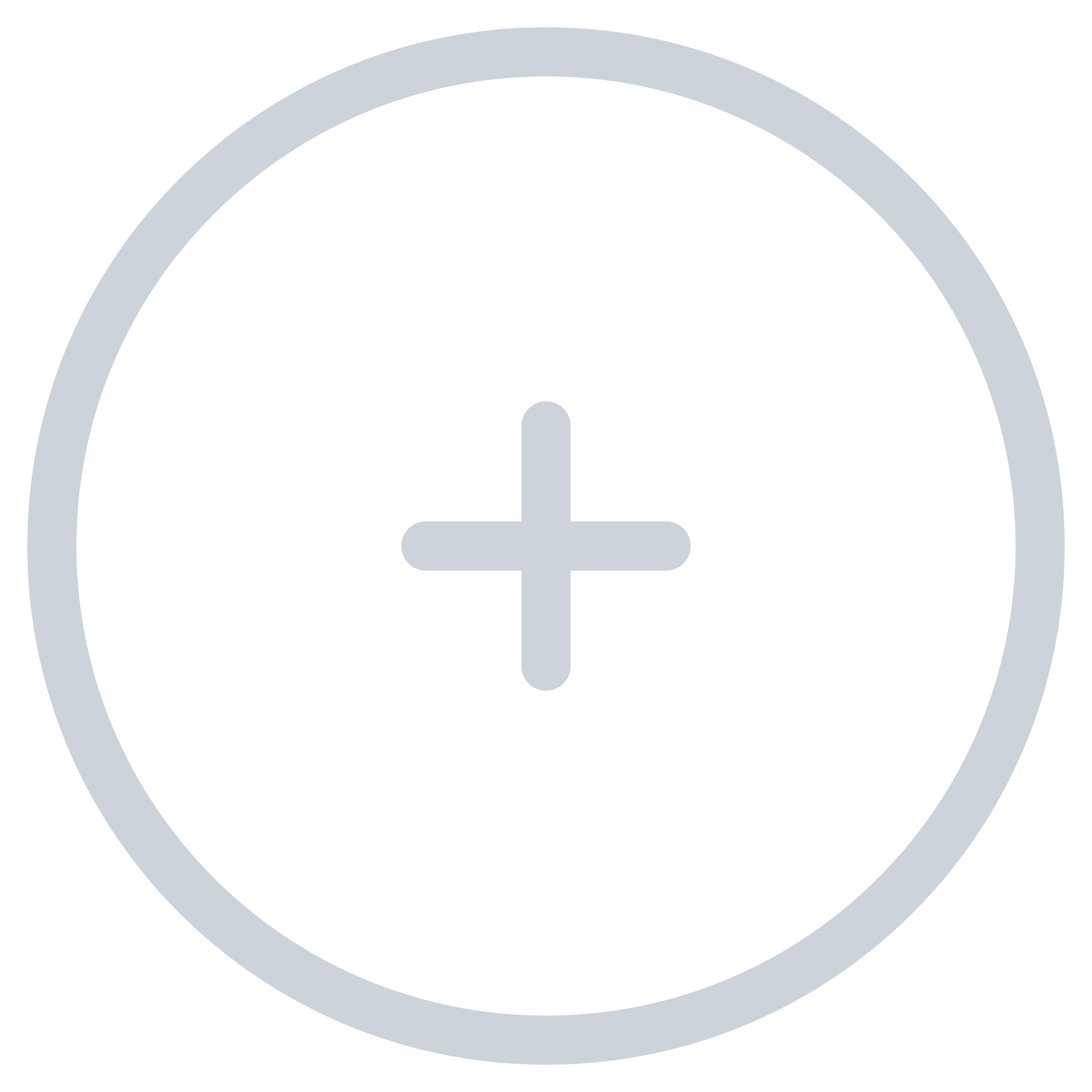 | **Moderate positive impact:** Statistically significant effect in >50% of studies or pooled meta-analysis  AND  Clinically significant effect on health in >50% of studies or pooled meta-analysis |
| 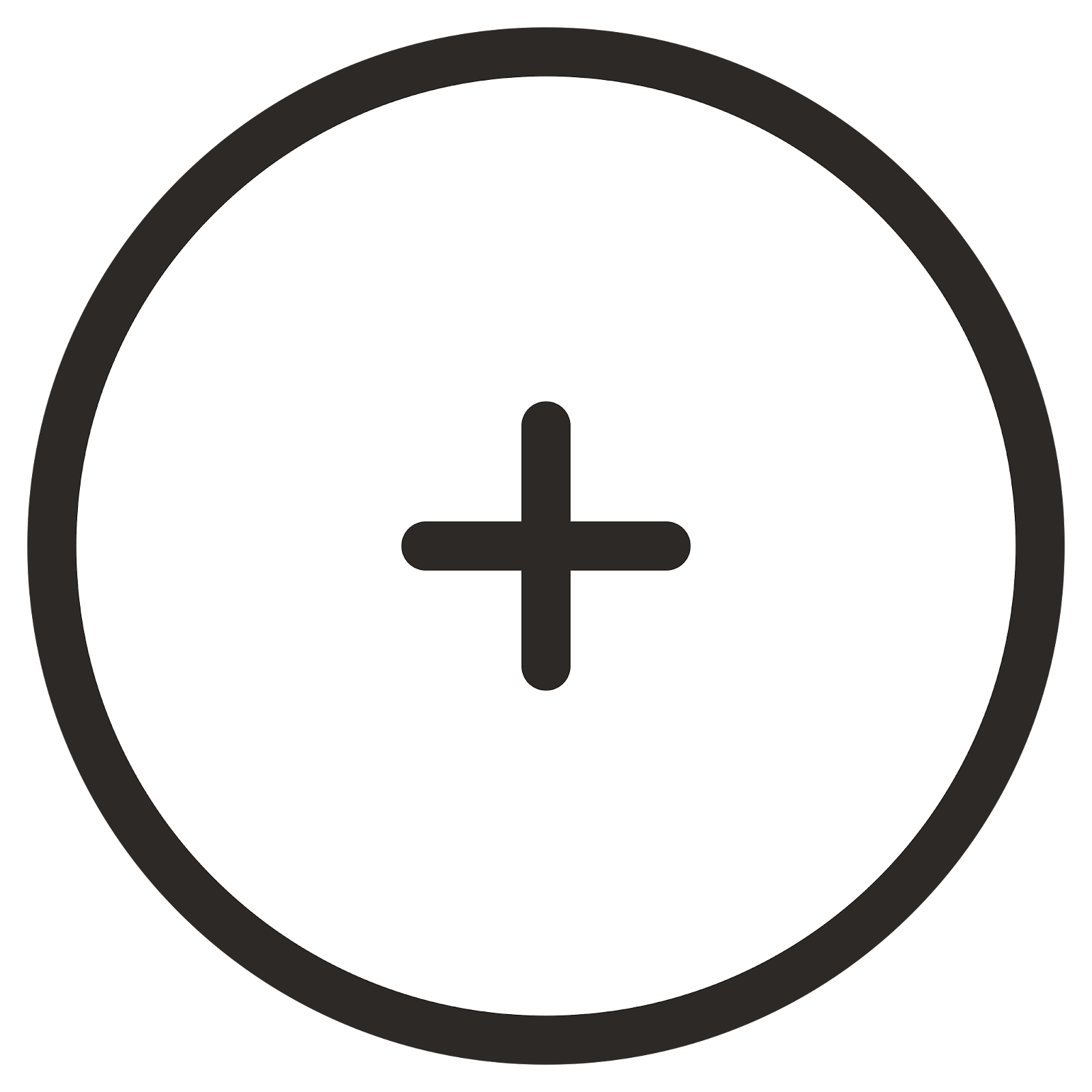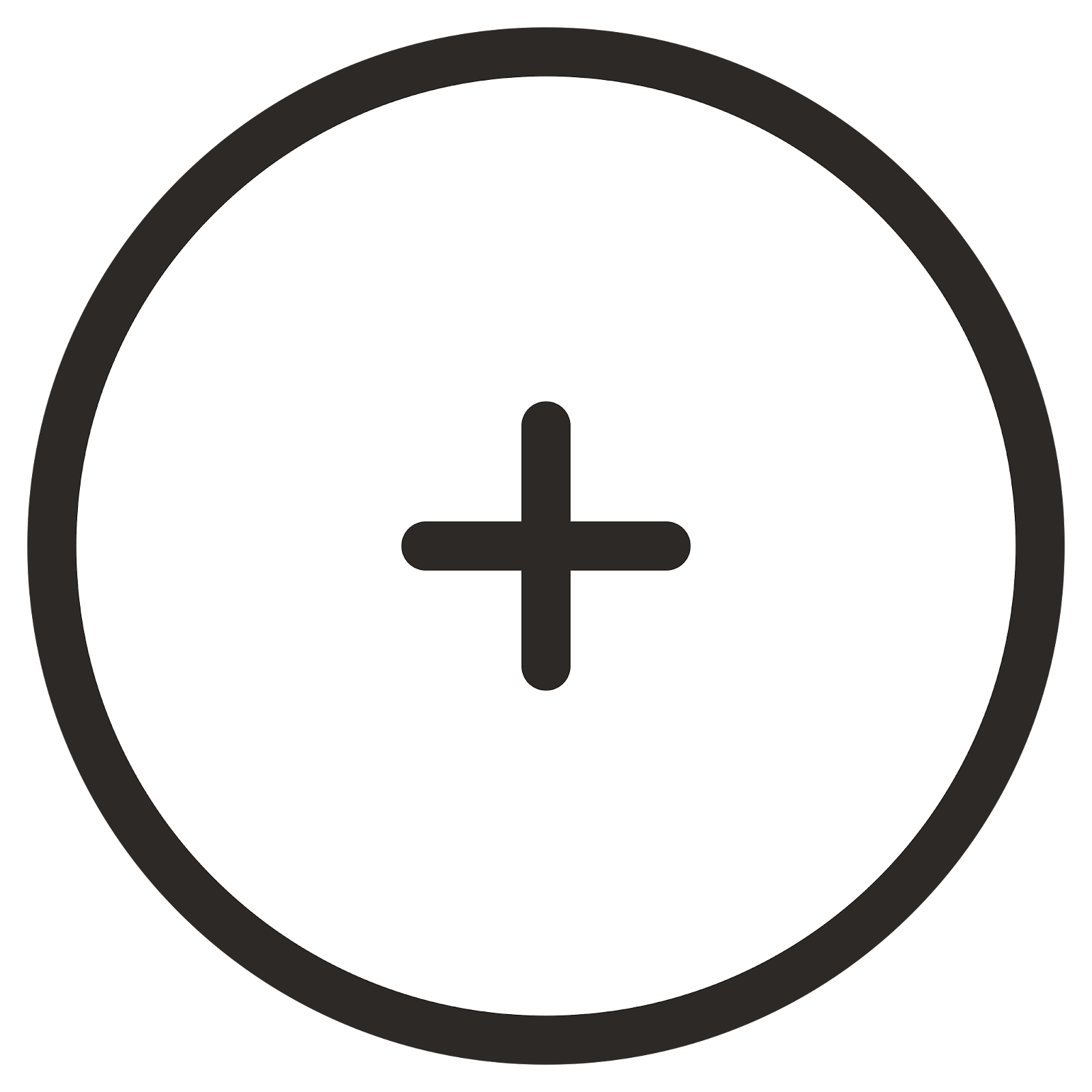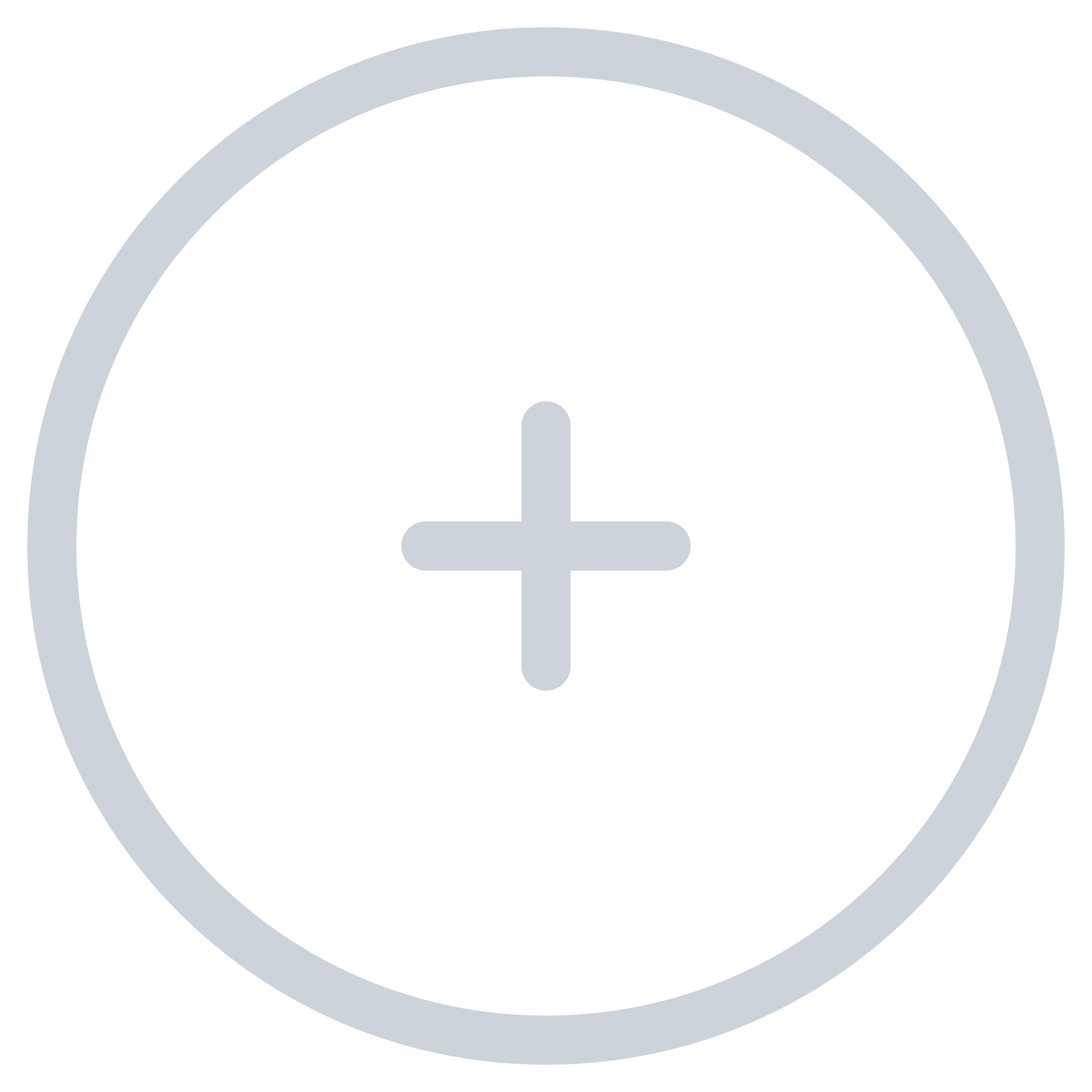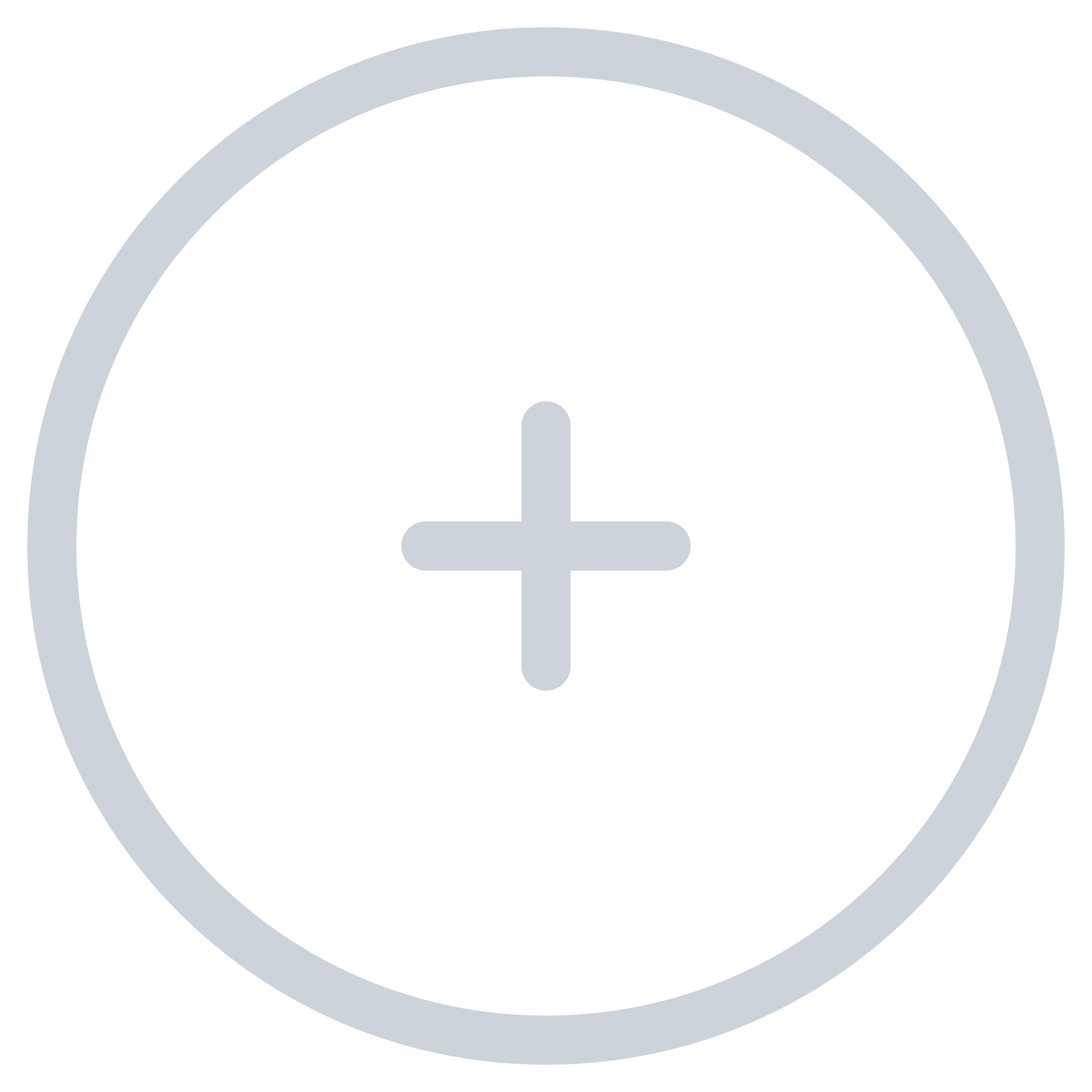 | **Mild positive impact:** Statistically significant effect in 50-25% of studies or pooled meta-analysis  OR  Clinically significant effect on health in 50-25% of studies or pooled meta-analysis |
| 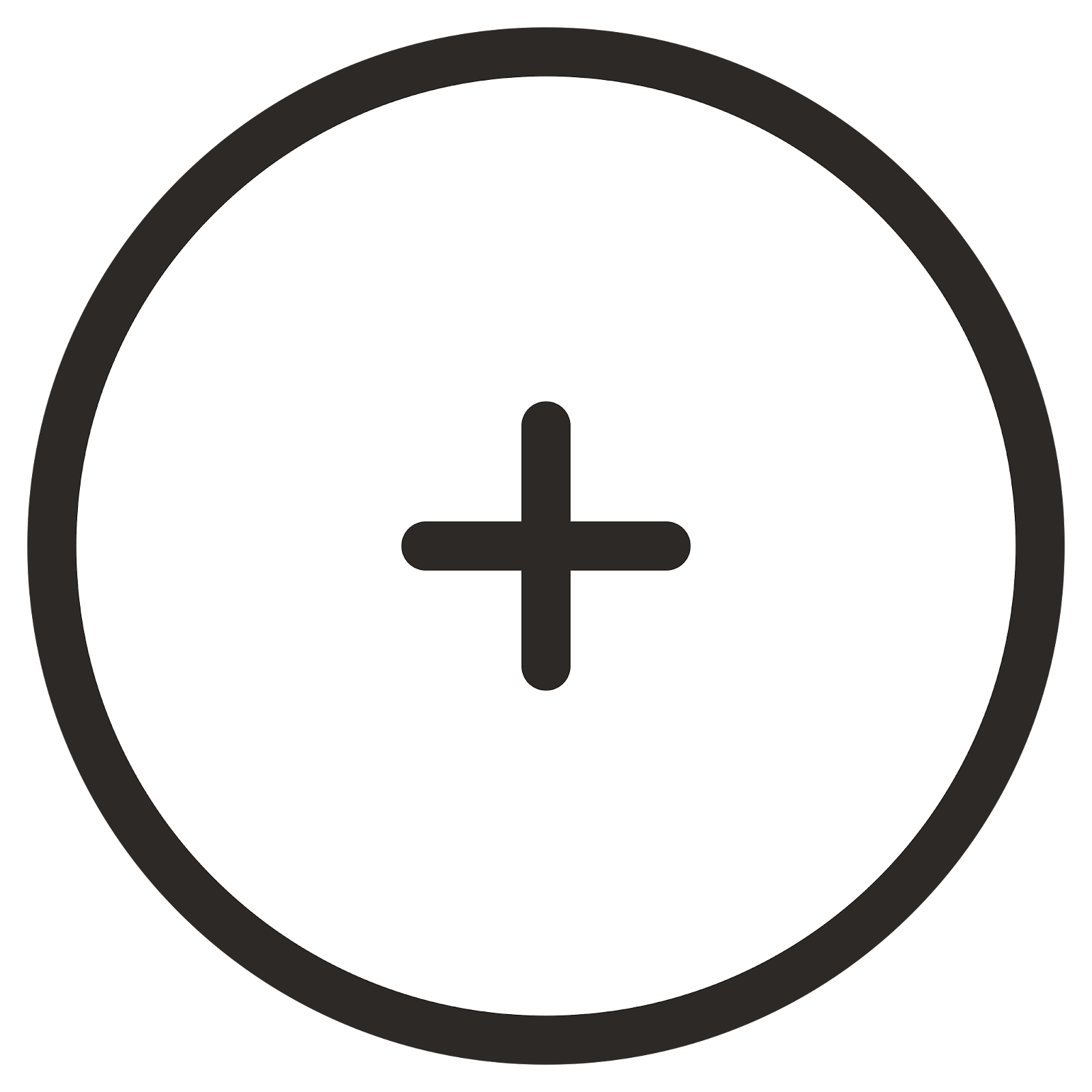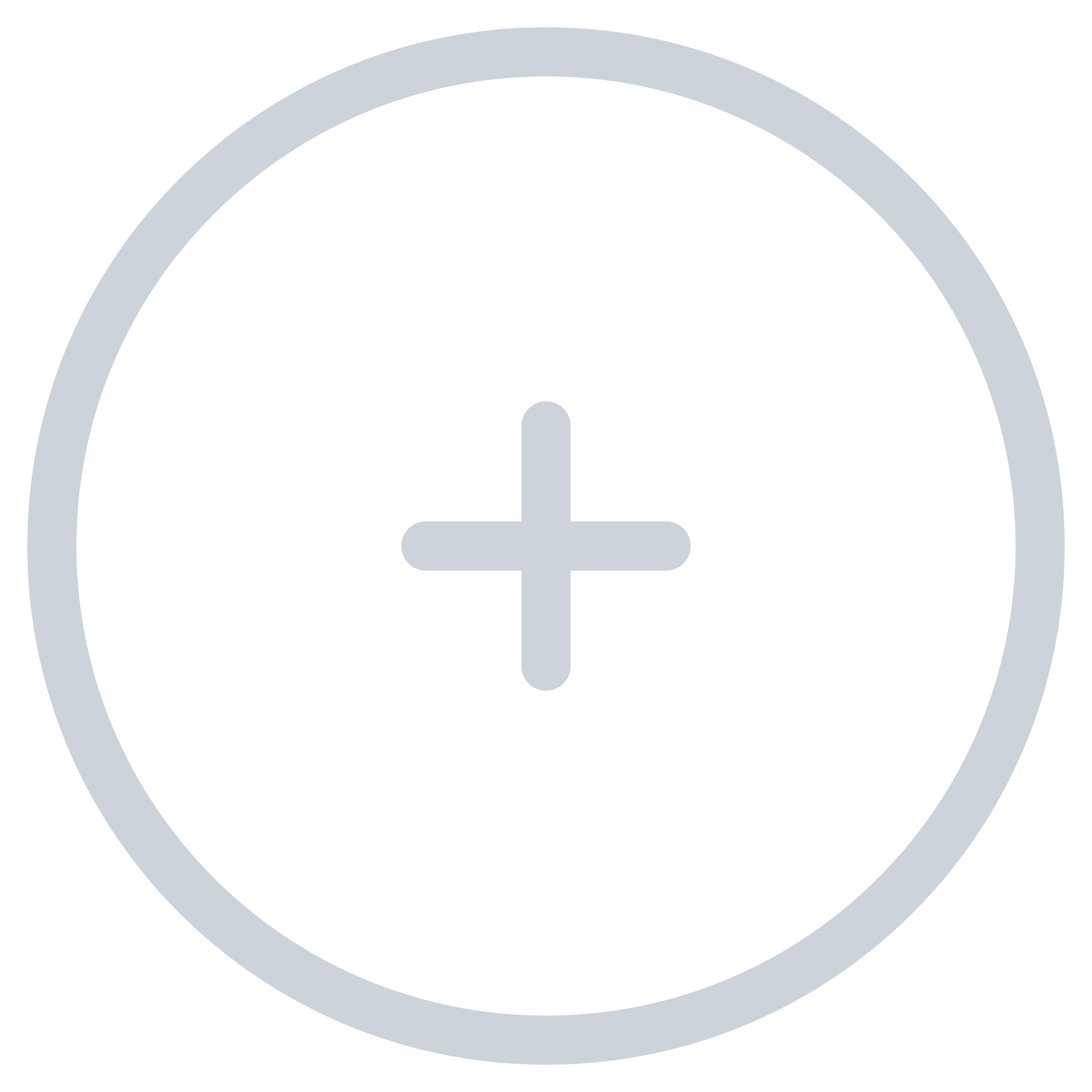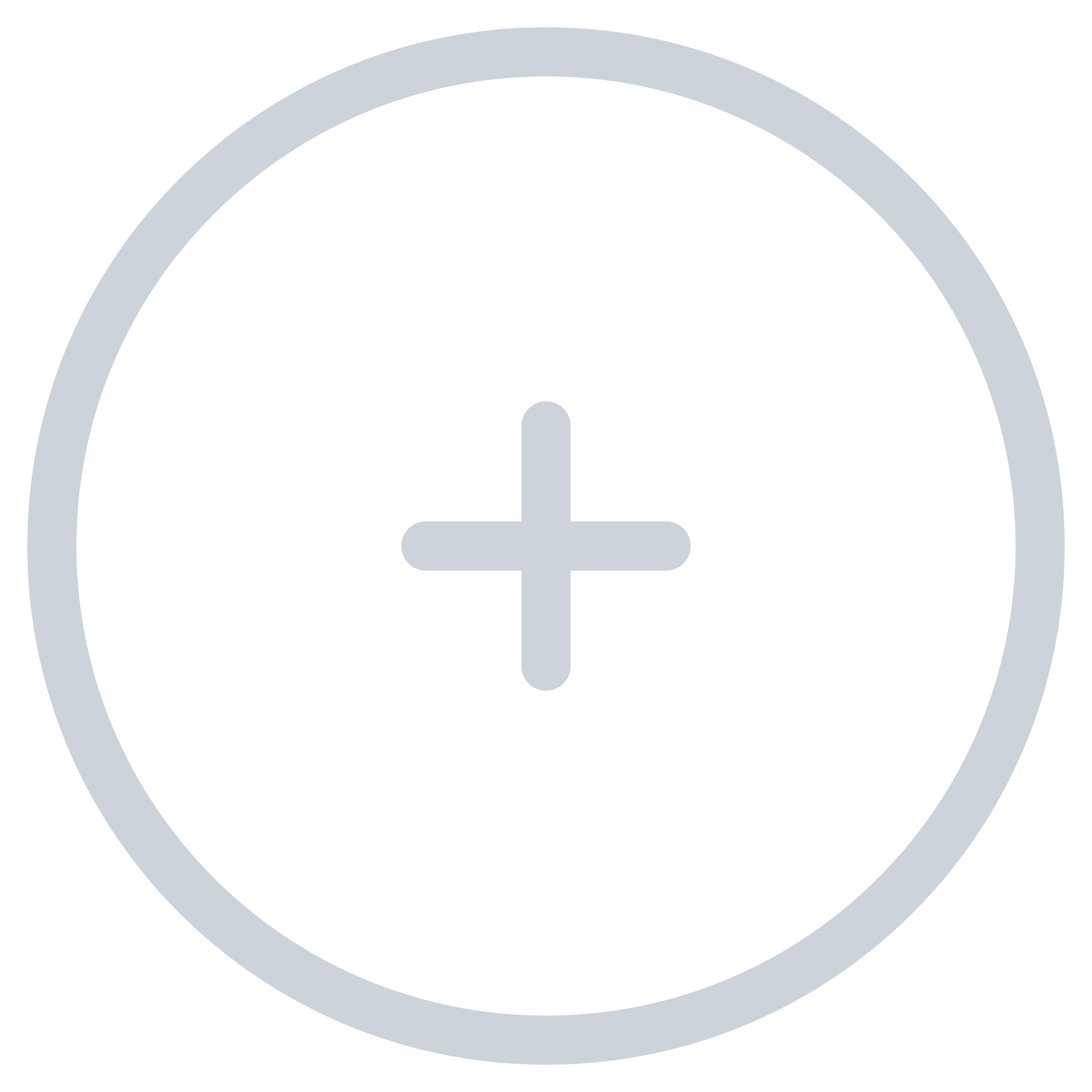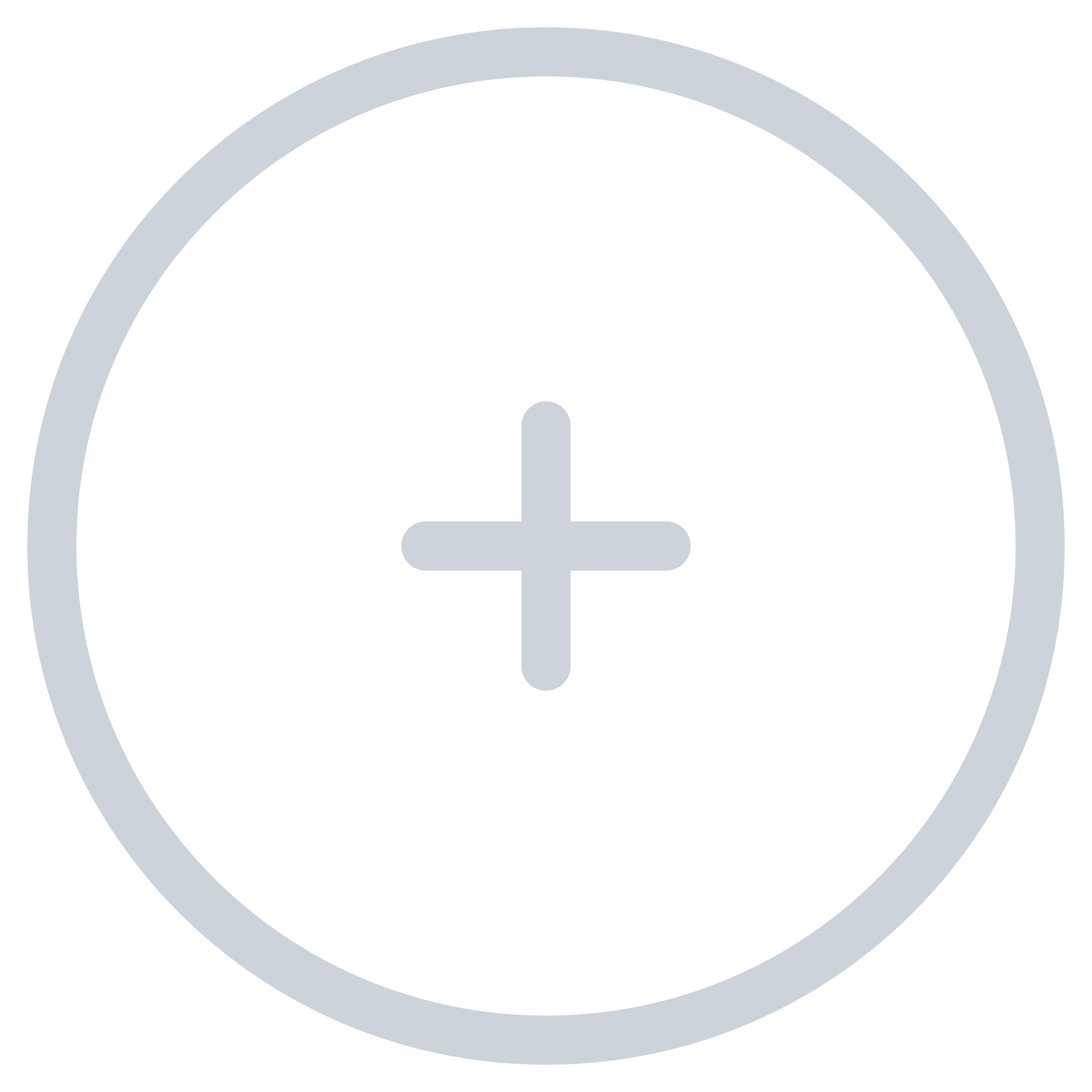 | **Low positive impact:** Statistically non-significant effect in >50% of studies or pooled meta-analysis  OR  Non clinically significant effect on health in >50% of studies or pooled meta-analysis |
| 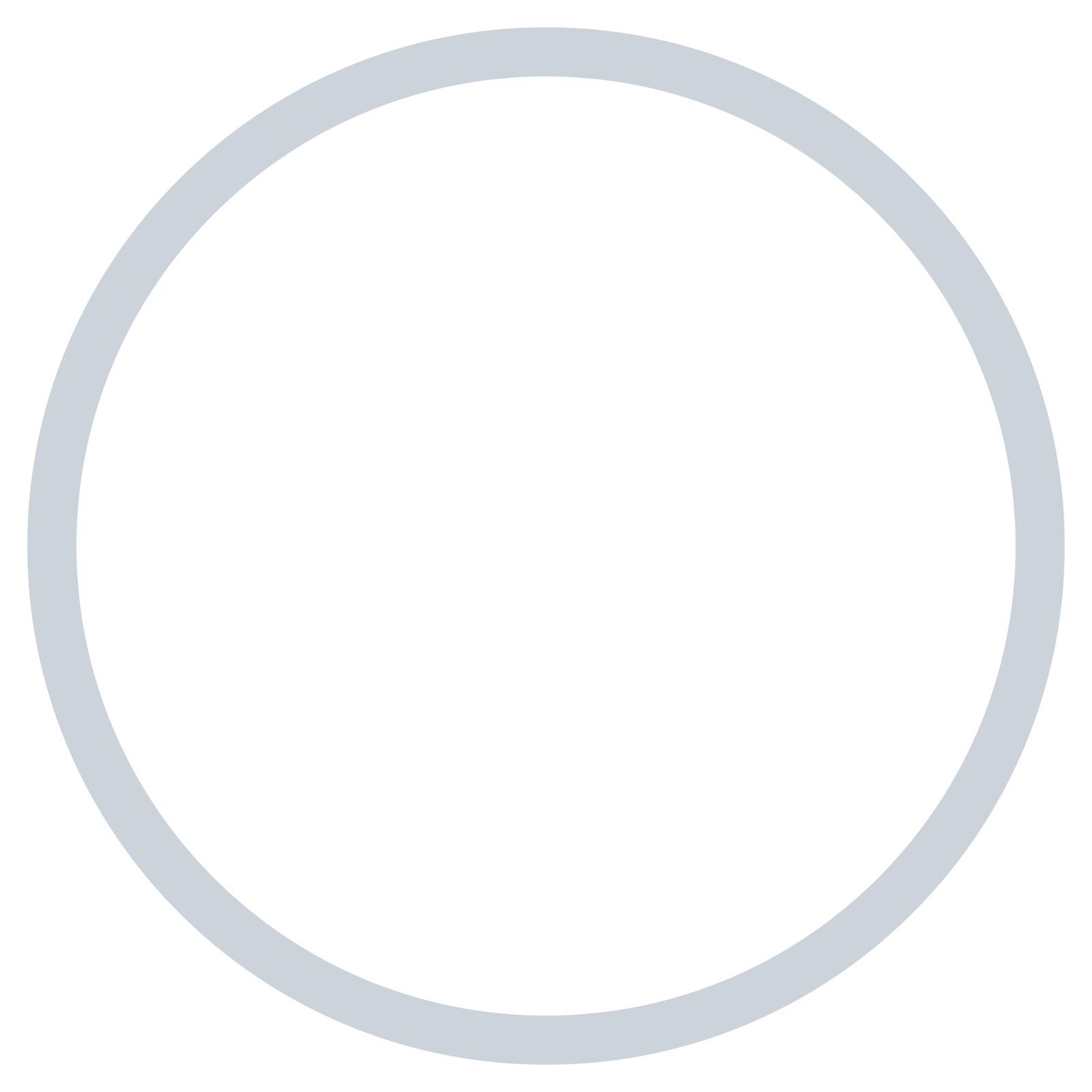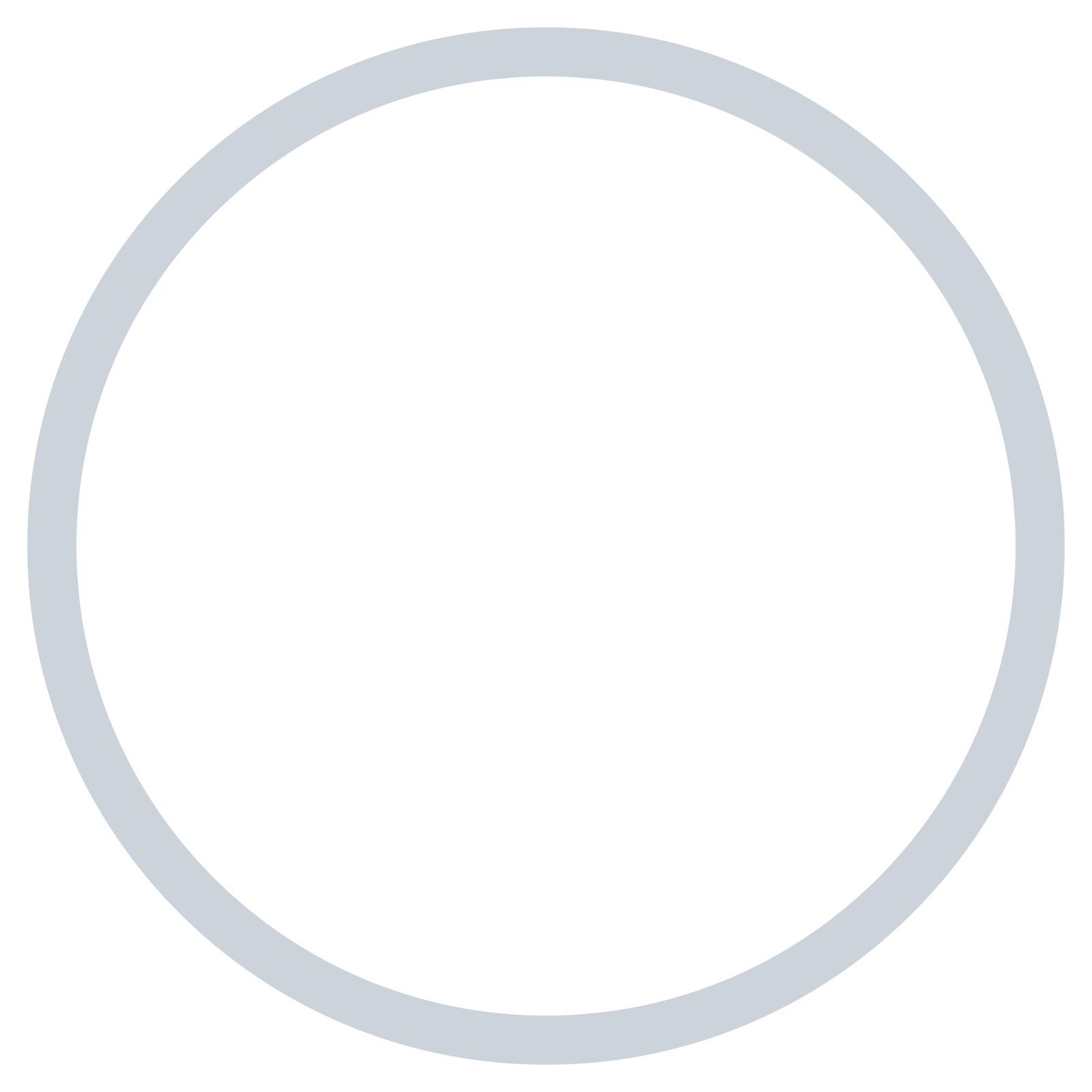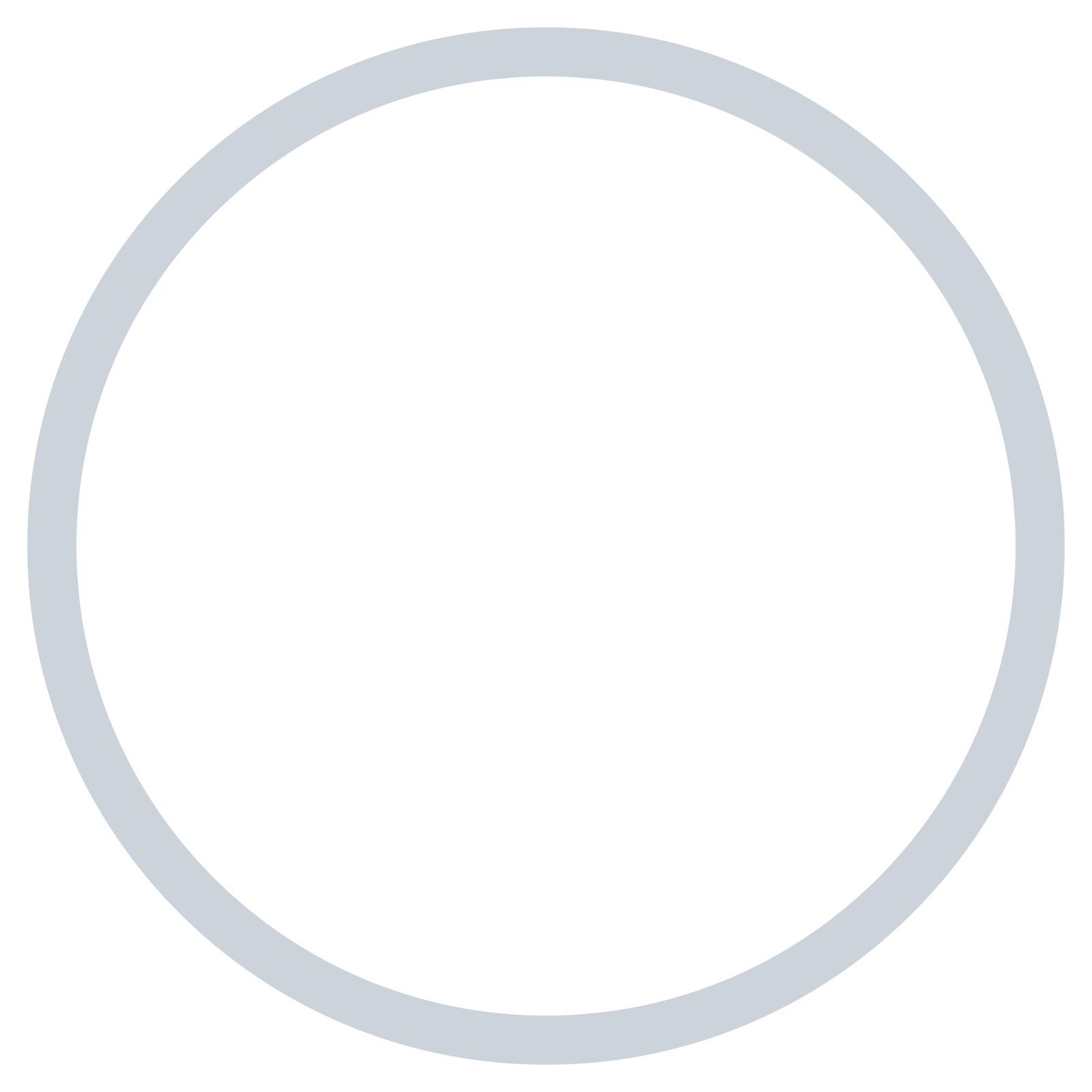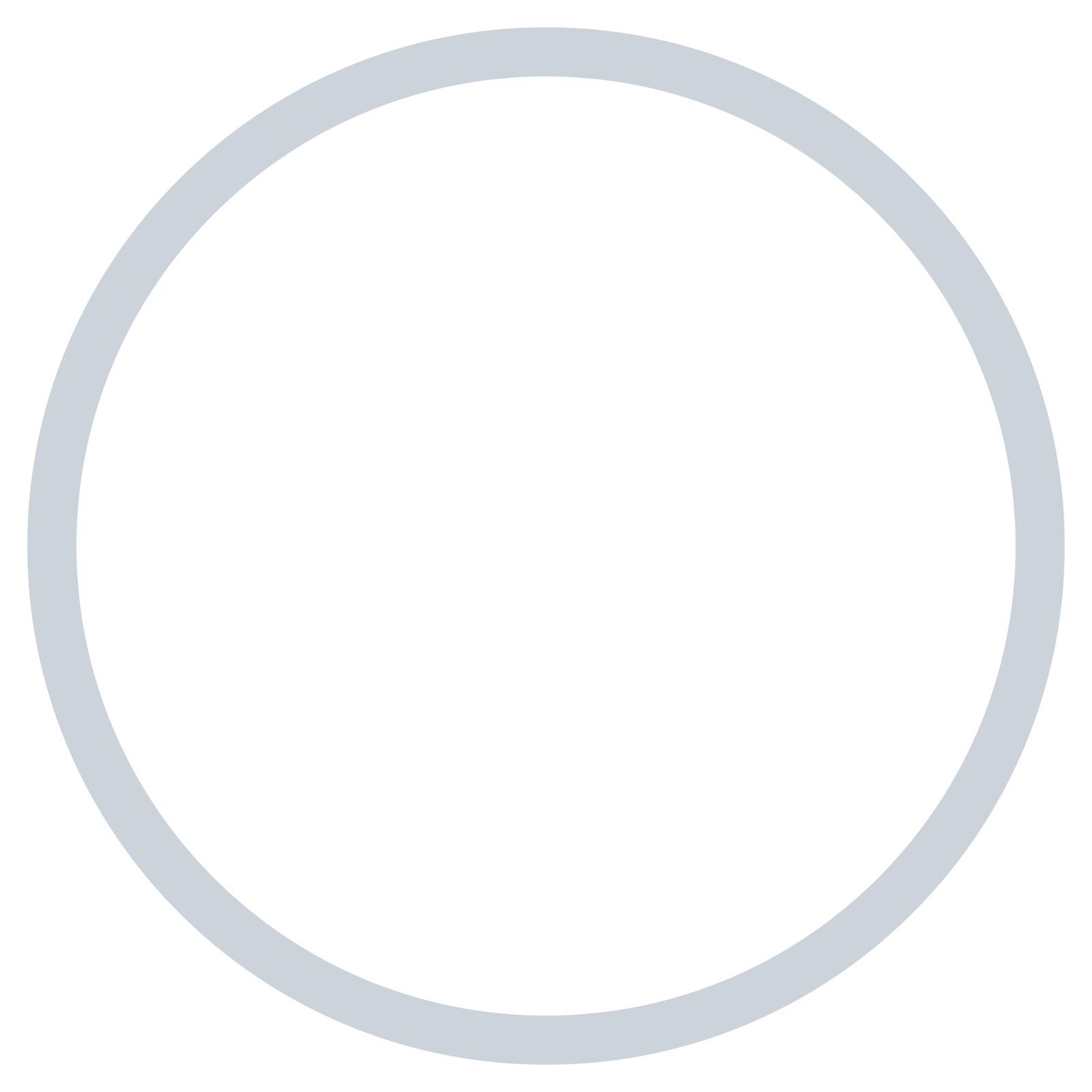 | **Uncertain:** Statistically non-significant effect in <50% of studies or pooled meta-analysis  **AND**  Non clinically significant effect on health in <50% of studies or in pooled meta-analysis |
| 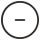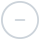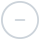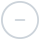 | **Low negative impact:** Statistically non-significant negative effect in >50% of studies or pooled meta-analysis  OR  Non-significant adverse effects on health in >50% of studies or pooled meta-analysis |
| 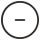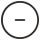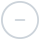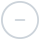 | **Mild negative impact:** Statistically significant negative effect in 50-25% of studies or pooled meta-analysis  OR  Significant adverse effects on health in 50-25% of studies or pooled meta-analysis |
| 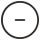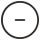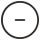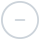 | **Moderate negative impact:** Statistically significant negative effect in >50% of studies or pooled meta-analysis  AND  Significant adverse effect on health in >50% of studies or pooled meta-analysis |
| 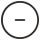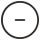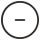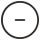 | **Strong negative impact:** Statistically significant negative effect in >75% of studies or pooled meta-analysis  AND  Significant adverse effect on health in >75% of studies or pooled meta-analysis |

*These parameters are subjective but use quartiles to categorise the included studies and will be reviewed on an ongoing basis.

##

## **What do we mean by ‘resources’?**

Resources are the collective requirement for funding, materials, labour, time or other assets required to deliver an urban intervention or the financial impact of the urban exposure on the individual, community, city or organisation. The judgments are subjective and therefore require integrating with the local context, which may affect the resources required.

| **Score** | **Parameters *** |
| --- | --- |
| 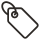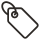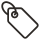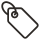 | **High resources:** >75% of included reviews reported resource requirements, and >50% were ranked as high |
| 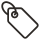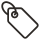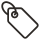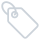 | **Moderate resources:** >50% of included reviews reported resource requirements, and >50% were ranked as moderate |
| 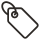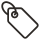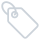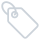 | **Low resources:** 50-25% of included reviews reported resource requirements, and >50% were ranked as low |
| 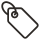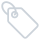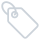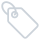 | **Very low resources:** 25% of included reviews reported resource requirements, and >50% were ranked as very low |
| 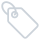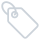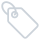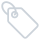 | **Uncertain:** >75% of included reviews did not report information regarding the cost |

*Detailed explanations of the resource implications are summarised on each toolkit page based on the evidence provided in the reviews. These parameters are subjective but use quartiles to categorise the included studies and will be reviewed on an ongoing basis.

## **How do we judge the quality of the ‘evidence’?**

The quality of the evidence reflects the rigour of research and helps attribute confidence in the results. Well-conducted evidence reviews use “risk of bias” or quality assessments to grade the evidence.

To assess the quality of the evidence, we used an adapted version of [AMSTAR 2](https://www.bmj.com/content/358/bmj.j4008), a popular instrument for critically appraising reviews of healthcare interventions and the reported findings from the risk of bias or quality assessments.

There are seven critical domains of the AMSTAR 2 instrument; we used six of the domains depending on the review methods. For all studies included in our Toolkit, we used the following domains:

- protocol registration before the commencement of the review (item 2),
- adequacy of the literature search (item 4), and
- justification for excluding individual studies (item 7).

For all studies included in our Toolkit, we also extracted the following information:

- design of the review,
- design of the studies included in the review,
- dates of the search (to assess how up to date the evidence is),
- numbers and types of databases used to search for evidence, and
- other evidence included (e.g. grey literature or backward searching).

If the included studies did not conduct a risk of bias or quality assessment, then the quality of the evidence was automatically rated as ‘uncertain’. If the included review conducted a risk of bias or quality assessment, then a further two domains of AMSTAR 2 were used, including

- consideration of the risk of bias when interpreting the results of the review (item 13) and
- assessment of the presence and likely impact of publication bias (item 15).

If the included studies also conducted a meta-analysis, then item 11 of AMSTAR 2 was used to grade the appropriateness of meta-analytical methods. Item 9 of AMSTAR 2 was not used as this domain was relevant for individual studies rather than reviews.

| **Score** | **Parameters*** |
| --- | --- |
| 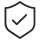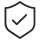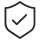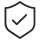 | **High quality:** >75% of included reviews conducted a risk of bias or quality assessment, and >50% of the assessment scores were ranked as low risk of bias or high-quality |
| 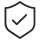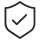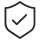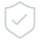 | **Moderate quality:** >50% of included reviews conducted a risk of bias or quality assessment, and >50% of the assessment scores were ranked as moderate risk of bias or quality |
| 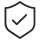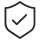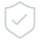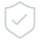 | **Low quality:** >50% of included reviews conducted a risk of bias or quality assessment, and >50% of the assessment scores were ranked as high-risk of bias or low quality |
| 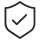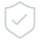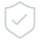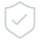 | **Very low quality: >**25% of included reviews conducted a risk of bias or quality assessment, and >50% of the assessment scores were ranked as very high-risk of bias or very low quality |
| 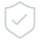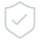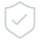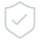 | **Uncertain:** <25% of included reviews conducted a risk of bias or quality assessment |

*These parameters are subjective, but use quartiles to categorise the included studies and will be reviewed on an ongoing basis.
